# Design, Synthesis and Structure-Activity Relationship Optimization of Lycorine Derivatives for HCV Inhibition

**DOI:** 10.1038/srep14972

**Published:** 2015-10-07

**Authors:** Duozhi Chen, Jieyun Cai, Junjun Cheng, Chenxu Jing, Junlin Yin, Jiandong Jiang, Zonggen Peng, Xiaojiang Hao

**Affiliations:** 1 State Key Laboratory of Phytochemistry and Plant Resources in West China, Kunming Institute of Botany, Chinese Academy of Sciences, Kunming 650201, China; 2 Laboratory of Antiviral Research, Institute of Medicinal Biotechnology, Chinese Academy of Medical Sciences/Peking Union Medical College, Beijing 100050, China; 3 College of Traditional Chinese Medicine, Yunnan University of Traditional Chinese Medicine, Kunming 650500, Yunnan, China

## Abstract

Lycorine is reported to be a multifunctional compound. We previously showed that lycorine is an HCV inhibitor with strong activity. Further research on the antivirus mechanism indicated that lycorine does not affect the enzymes that are indispensable to HCV replication but suppresses the expression of Hsc70 in the host cell to limit HCV replication. However, due to the cytotoxicity and apoptosis induction of lycorine, lycorine is unsafe to be a anti-HCV agent for clinical application. As a result of increasing interest, its structure was optimized for the first time and a novel series of lycorine derivatives was synthesized, all of which lost their cytotoxicity to different degrees. Structure-activity analysis of these compounds revealed that disubstitution on the free hydroxyl groups at C1 and C2 and/or degradation of the benzodioxole group would markedly reduce the cytotoxicity. Furthermore, an *α*, *β*-unsaturated ketone would improve the HCV inhibitory activity of lycorine. The C3-C4 double bond is crucial to the anti-HCV activity because hydrogenation of this double bond clearly weakened HCV inhibition.

Hepatitis C virus (HCV) is seriously harmful to human health and is one of the most common causes of chronic hepatitis worldwide^1^,^2^,^3^. Several potent HCV inhibitors have been developed^4^,^5^,^6^,^7^, but resistance to them has developed due to the high variability of HCV. There is therefore a need for alternative HCV drugs, which are in development^8^,^9^,^10^.

Lycorine (**1**, Fig. 1a) is a multifunctional benzylphenethylamine alkaloid^11^,^12^. We previously reported that it has good activity against HCV. Upon investigating the anti-HCV mechanisms of lycorine, we found that it works by down-regulating the level of intracellular Hsc70 protein (Fig. 1b,c) in a dose-dependent fashion, as shown by the positive correlation between them (Fig. 1d). Lycorine was formerly known as an antitumour agent. Previous reports showed that it exhibits good cytotoxicity and apoptosis induction activities in cancer cell lines^13^,^14^,^15^. As a result, although lycorine is clearly an inhibitor of HCV, it poses safety issues. We therefore investigated the structure-activity relationships of lycorine analogues to identify a related compound with comparable levels of HCV inhibition but less cytotoxicity. Here, we describe the structural optimization process of lycorine and present an evaluation of its derivatives’ anti-HCV effects and toxicity *in vitro*. We also analysed their structure-activity relationships (SAR).

## Results and Discussion

### Design and Synthesis of Lycorine Derivatives

**Figure 2:**
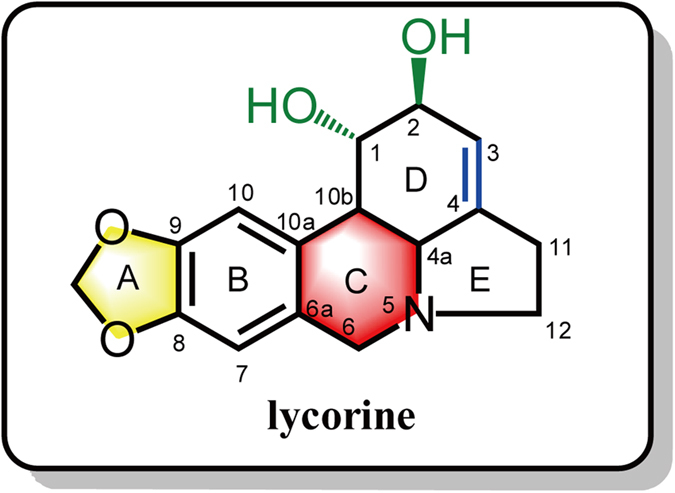
Regions of the lycorine skeleton that were targeted for modification. The C-1 and C-2 hydroxyl groups (green labelled), the double bond between C-3 and C-4 (blue labelled); the C-ring of the structure (red labelled) and the benzodioxole group (yellow labelled).

As shown in Fig. 2, our initial attempts at modifying the structure of **1** focused on manipulating the C-1 and C-2 substituents (green-labelled) and the C3-C4 double bond (blue-labelled). In addition, we investigated the effects of modifying the properties of the nitrogen atom at the 5-position of the lycorine skeleton (red-labelled) and the benzodioxole group (yellow-labelled). Fifteen lycorine derivatives were ultimately synthesized, bearing various combinations of these modifications.

**Figure 3:**
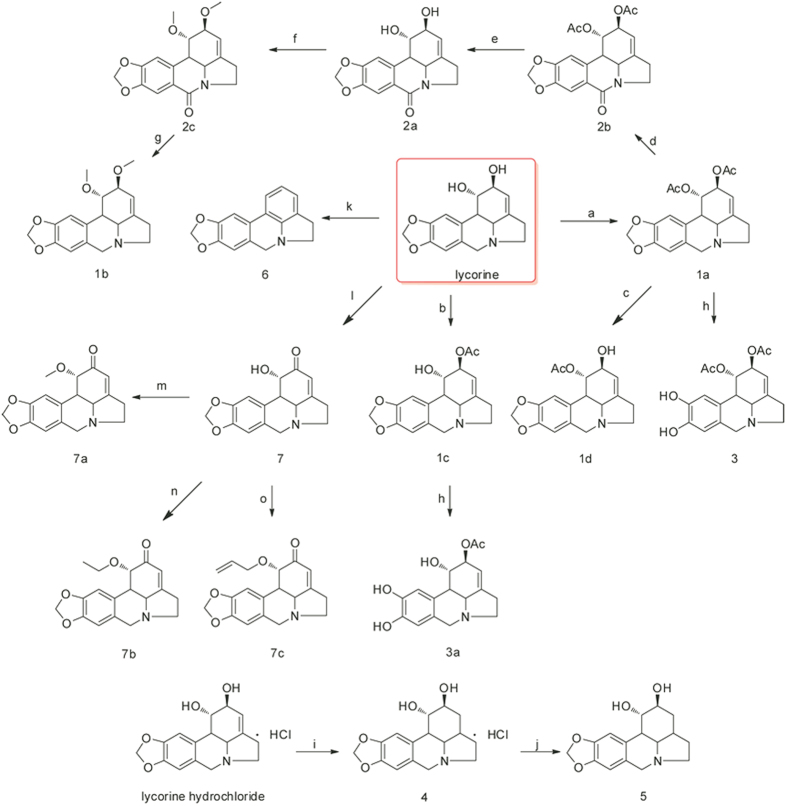
Synthesis of lycorine derivatives. Reagents and conditions: (a) Ac_2_O (3.0 eq), DMAP, py, r.t., 8 h, 78%; (b) Ac_2_O (1.0 eq), py, r.t., 2 h, 40%; (c) 10% HCl, 60 °C, 1.5 h, 40%; (d) PhIO, NBu_4_I, CH_3_CN/H_2_O 9:1, r.t., 6 h, 75%; (e) K_2_CO_3_, MeOH/H_2_O 9:1, 60 °C, 4 h, 90%; (f) NaH, MeI, THF, r.t., 24 h, 80%; (g) LAH, THF, −78 °C, 6 h, 75%; (h) BBr_3_, CH_2_Cl_2_, −78 °C, 6 h, 75% for 3, 60% for 3a; (i) 10% Pd/C, H_2_, H_2_O, r.t., 24 h; (j) NaOH, H_2_O, 95%; (k) Burgess reagent, DMF, 50 °C, 2 h, 70%; (l) Jones reagent, r.t., 8 h, 60%; (m) Ac_2_O, py, 60 °C, 8 h, 72%; (n) propionic anhydride, py, 60 °C, 8 h, 65%; (o) Butyric anhydride, py, 60 °C, 12 h, 70%.

The first lycorine derivatives were prepared by acetylating the C-1 and C-2 hydroxyl groups of the parent compound. Controlled acetylation was achieved by varying the quantities of the reagents used (acetic anhydride and DMAP) and the reaction time, yielding 1, 2-di-O-acetyllycorine (**1a**) and 2-O-acetyllycorine (**1c**). Compound **1a** was then hydrolysed to obtain 2-O-acetyllycorine (**1d**)^13^,^16^. Compounds **1c** and **1d** bear similar structural features, although they differ in having an acetoxy group at the C-1 or C-2 position, respectively. This difference was confirmed by comparing the ^1^H and ^13^C NMR data with relevant references^13^,^16^. Compound **1a** was first oxidized using PhIO and NBu_4_I to afford 1,2-diacetyl-6-carbonyllycorine (**2b**). The acetyl groups of **2b** were then cleaved to yield **2a**, which was further etherified with methyl iodide to give **2c**^16^. Subsequent reduction of the C-6 carbonyl of **2c** afforded 1, 2-dimethoxylycorine (**1b**). The benzodioxole group of **1a** and **1c** was degraded using BBr_3_ to afford compounds **3** and **3a**, respectively^16^. In addition, lycorine was selectively oxidized by Jones agents to afford **7**, followed by etherification to get compounds **7a**–**c**. Lycorine hydrochloride was transformed to dihydrolycorine hydrochloride (**4**) by hydrogenation, followed by reaction with NaOH to furnish compound **5** (Fig. 3).

### The Anti-HCV Activity of Lycorine Derivatives

All 15 analogues were examined to determine their effects on HCV replication in Huh 7.5 cells (EC_50_), and their cytotoxicity (CC_50_) was assessed *in vitro* to determine their selective index (SI) values (Table 1) using Cyclopenta [*c*]pyrrole-1-carboxamide (VX-950), which is an inhibitor of the HCV NS3/4A protease, as a positive control^17^. The data indicate that these compounds lost their cytotoxicity to different degrees, and some of these compounds, such as **1a** and **3**, exhibited reduced cytotoxicity while possessing levels of anti-HCV activity similar to that of lycorine.

### SAR Analysis of the Anti-HCV Effects of Lycorine Derivatives

**Figure 4:**
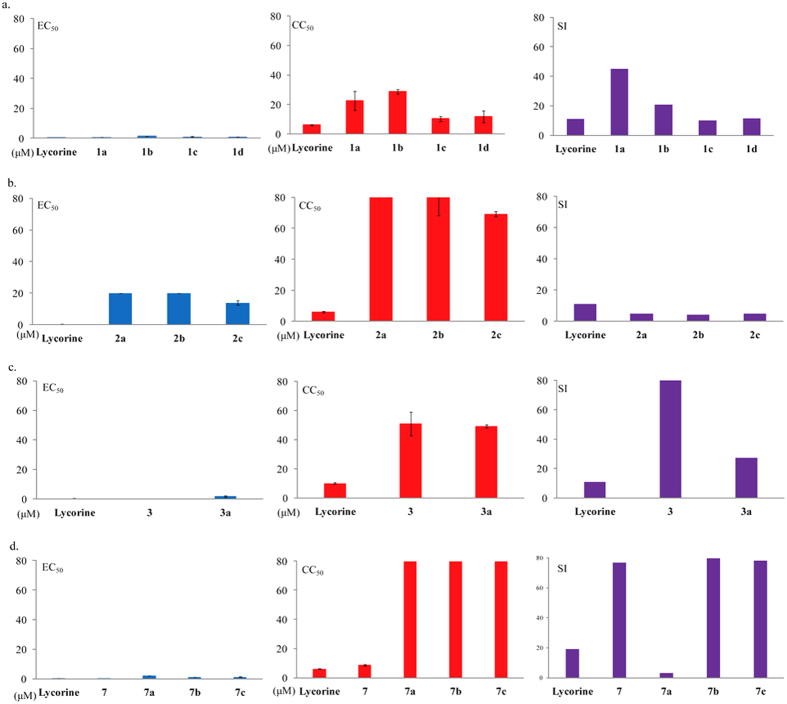
SAR analysis of lycorine derivatives. (**a**) Disubstitution, especially the diacetylation of the free hydroxyl groups at C-1, C-2, should markedly reduce the cytotoxicity; (**b**) Oxidization of the basic nitrogen atom of lycorine reduces its anti-HCV activity; (**c**) Degradation of the benzodioxole group reduces the cytotoxicity. The SI value of 3 is greater than 50. (**d**) An *α*, *β*-unsaturated ketone would improve the HCV inhibitory activity of lycorine. The CC_50_ value of compound 2a, 2b, 7a, 7b and 7c are higher than 80 *μ*M. The SI values of compound 3 and 7b are higher than 80. All mean and standard deviation data were obtained from quintuplicate experiments (N = 5).

The SAR study initially focused on the effects of modifying the C-1 and C-2 hydroxyl groups of lycorine by examining compounds **1a**–**d**. The disubstituted derivative **1a** and lycorine exhibited similar levels of anti-HCV activity, but the other three analogues, **1b**, **1c** and **1d**, exhibited weaker anti-HCV activities, to varying degrees. These results suggest that diacetyl derivatives have stronger anti-HCV activity than monoacetylated or dimethylated ones, so the hydroxyl groups at C-1 and C-2 could be crucial to the cytotoxicity of lycorine (Fig. 4a).

The nitrogen atoms in alkaloids are known to have profound effects on their biological activities. The oxidized lycorine derivatives **2a**–**c** were therefore prepared to determine how the conversion of the parent compound’s basic nitrogen into an amide would affect its biological activity. All three derivatives had substantially lower anti-HCV activity than lycorine itself, regardless of their C-1 and C-2 substituents. The basic nitrogen at the N-5 position thus seems be very important to the anti-HCV activity (Fig. 4b).

Notably, the two derivatives with a degraded benzodioxole group (**3** and **3a**) were substantially less cytotoxic than the parent compounds. Compared to **1a**, compound **3** lost a large portion of its cytotoxicity, while its anti-HCV activity remained nearly constant. A similar phenomenon can be observed in the comparisons of **1a** with **3a**. It appears that the benzodioxole group is another key site of cytotoxicity whose removal has little effect on HCV inhibition (Fig. 4c).

*α*, *β*-unsaturated ketones are widely considered to be crucial to the bioactivities of compounds, so we designed lycorine derivatives with an *α*, *β*-unsaturated ketone substructure (compounds **7**, **7a**, **7b** and **7c**) by the oxidation of the hydroxyl at the C-2 position. An anti-HCV bioassay indicated that the unsaturated ketone could indeed enhance the anti-HCV activity of compounds, such as **7**. However, when the hydroxyl at the C-1 position was substituted by a methyl (**7a**), ethyl (**7b**) or allyl (**7c**) group, the cytotoxicity decreased and the anti-HCV activity was degraded (Fig. 4d). It can be inferred that an *α*, *β*-unsaturated ketone would improve the HCV inhibitory activity of lycorine.

The importance of the C3-C4 double bond was then investigated. Hydrogenation of this double bond noticeably alters the electron distribution within ring D of lycorine and may thereby affect its anti-HCV activity. The comparison of lycorine and compound **5** indicates that a compound without the double bond, such as **5**, does not show anti-HCV activity. It thus seems that the C3-C4 double bond is crucial to the anti-HCV activity. The aromatization of ring D (compound **6**) also causes a loss of the anti-HCV activity of lycorine.

### Evaluation of the Apoptosis Induction Activity of Lycorine Derivatives

**Figure 5:**
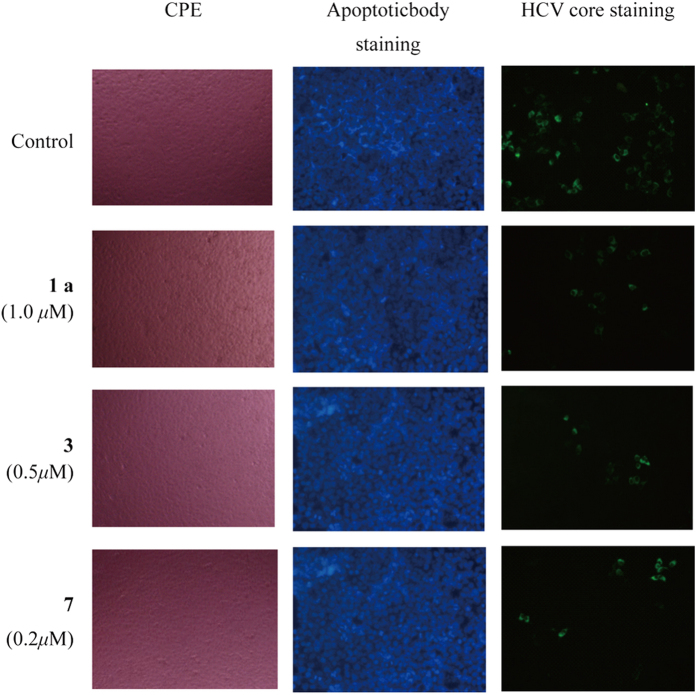
Apoptosis induction activity of lycorine derivatives. The compounds showed no cytotoxicity with CPE or apoptoticbody staining method, but with anti-HCV activity at the dose of 2 times of EC_50_ in HCV-infected Huh 7.5 cells.

As mentioned above, some scientists reported that lycorine exhibits significant antitumour activity correlated with apoptosis^15^. We analyzed the anti-HCV activities of modified lycorine using qRT-PCR under the circumstance with the cells no cytotoxicity judged with CPE method and confirmed with MTT staining method after 72 hours treatment with derivatives. If there is no cytotoxicity with CPE or MTT method after 72 hours treatment with compounds, there is no apoptosis which will more or less show cytotoxicity that can be detected with those methods. To confirm this speculate, we added three of good HCV inhibitors **1a**, **3** and **7** at the concentration of 2 times of EC_50_ into HCV-infected Huh 7.5 cells. At that dose, they showed no cytotoxicity with CPE method (Lane CPE) and also no apoptosis confirmed with apoptoticbody staining method (Lane Apoptoticbody staining). But, they still showed strongly anti-HCV activity (Lane HCV core staining). These results indicated that lycorine derivatives could not induce Huh 7.5 cell apoptosis (Fig. 5).

## Conclusions

The cytotoxic lycorine could become a safe and efficient HCV inhibitor through appropriate modifications. According to the processes of the structure-activity relationship optimization of lycorine derivatives, we conclude that the C3-C4 double bond and the basic nitrogen at N-5 position are crucial to the anti-HCV activity. The benzodioxole group and the hydroxyl groups at C-1 and C-2 seem to be very important to the cytotoxicity. The disubstitution, especially the diacetylation of the free hydroxyl groups at C-1 and C-2 and/or the degradation of the benzodioxole group, should markedly reduce the cytotoxicity of the lycorine derivatives. However, this is accompanied by a variable reduction in the anti-HCV activity. The addition of an *α*, *β*-unsaturated ketone would improve the HCV inhibitory activity. As the C3-C4 double bond is crucial to the anti-HCV activity, the hydrogenation of this double bond would clearly weak the HCV inhibition. These findings provide constructive guidance for further structural modifications of lycorine.

## Methods

### Chemical

#### Reaction procedures of lycorine derivatives

##### 1,2-Di-O-Acetyllycorine (1a)

Lycorine (30 mg 0.1 mmol) was added to a solution of pyridine (3 mL), Ac_2_O (30 mg, 0.3 mmol), and DMAP (20 mg). The solution was stirred at room temperature for 8 h under N_2_ and then poured into ice-cold water (50 mL). The mixture was extracted with EtOAc (30 mL) twice and washed with saturated NaHCO_3_ followed by brine and then concentrated after separation by silica gel column chromatography to afford compound **1a** with a yield of 78%.

##### 2-O-Acetyllycorine (1c)

Lycorine (33 mg 0.1 mmol) was added into a solution of pyridine (3 mL) and Ac_2_O (8 mg, 0.08 mmol). The solution was stirred at room temperature for 2 h under N_2_ and then poured into ice-cold water (50 mL) with vigorous stirring. The mixture was extracted with EtOAc (30 mL) twice, washed with saturated NaHCO_3_ and brine, and then concentrated following separation by silica gel column chromatography to afford compound **1c** with a yield of 40%.

##### 1-O-Acetyllycorine (1d)

A solution of **1a** (37 mg, 0.1 mmol) in 5 mL of 10% HCl was heated at 60 °C for 1.5 h. Then, the reaction mixture was treated with NaHCO_3_ solution until a basic pH was reached and extracted several times with CH_2_Cl_2_. The organic phases were dried over MgSO_4_, concentrated and purified by silica gel column chromatography to obtain 1-O-acetyllycorine (**1d)** (18 mg, 40%).

##### 1,2-Di-O-Acetyl-6-carbonyl-lycorine (2b)

Tetrabutylammonium iodide (TBAI, 37 mg, 0.1 mmol) and ISB (308 mg, 1.4 mmol) were dissolved in MeCN/H_2_O 9:1 (10 mL). Compound **1a** (0.5 mmol) was subsequently added to the mixture, which was stirred for 6 h at room temperature and then diluted with toluene (100 mL). The organic layer was washed with sodium hyposulphite solution (2 × 10 mL) and saturated brine (2 × 10 mL). It was then concentrated under reduced pressure to yield the crude product, which was purified by silica gel column chromatography to afford 1,2-di-O-acetyl-6-carbonyl-lycorine (**2b**) with a yield of 75%.

##### 6-Carbonyllycorine (2a)

A solution of compound **2b** (34.3 mg, 0.1 mmol) and CH_3_OH/H_2_O (9:1, 10 mL) was added to K_2_CO_3_ (41.4 mg, 0.3 mmol). The mixture was stirred for 4 h at 60 °C and then diluted with EtOAc (30 mL). The organic layer was washed with NH_4_Cl solution (2 × 10 mL) and saturated brine (2 × 10 mL) and concentrated following separation by silica gel column chromatography to afford 6-carbonyllycorine (**2a**) with a yield of 90%.

##### 1,2-Di-O-Methyl-6-carbonyllycorine (2c)

Compound **2a** (30 mg, 0.1 mmol) was dissolved in dry THF (10 mL), after which NaH (50 mg, 2 mmol) and CH_3_I (1 mmol) were added. The mixture was stirred at room temperature for 24 h and then quenched with water (50 mL) in an ice bath. The reaction solution was evaporated to remove THF and extracted with CH_2_Cl_2_ (30 mL) twice. The organic layer was washed with saturated NaHCO_3_ and then brine, dried over MgSO_4_, filtered, and concentrated. The residue was purified by column chromatography to afford 1,2-di-O-methyl-6-carbonyllycorine (**2c**), with a yield of 80%.

##### 1,2-Di-O-Methyllycorine (1b)

Compound **2c** (33 mg, 0.1 mmol) was dissolved in THF (5 mL). The reaction solution was then cooled to −78 °C, and LiAlH_4_ (7.6 mg, 0.2 mmol) was added. The mixture was stirred for 2 h and diluted in H_2_O (10 mL). The solution was extracted with CH_2_Cl_2_ (15 mL) twice. The organic layer was washed with brine, concentrated, and then purified by column chromatography to give 1,2-di-O-methyllycorine (**1b**), with a yield of 80%.

##### 1,2-Di-O-Acetyllycorine-8,9-diphenol (3)

Compound **1a** (70 mg, 0.2 mmol) was dissolved in 10 mL CH_2_Cl_2_. The reaction solution was then cooled to −78 °C, and BBr_3_ (120 *μ*L, 0.3 mmol) was added. The mixture was then stirred for 6 h and diluted in 50 mL saturated NaHCO_3_, after which it was extracted twice with CH_2_Cl_2_ (20 mL). The organic layer was then washed with brine and concentrated. The residue was purified by column chromatography using chloroform-methanol (15:1) as the eluent to give 1,2-di-O-acetyllycorine-8,9-diphenol (**3**) as a pale yellow powder (49.5 mg, yield 75%).

##### 2-O-Acetyllycorine-8,9-diphenol (3a)

Compound **3a** was synthesized from **1c** (66 mg, 0.02 mmol) by the same procedure as **3**. The crude product was purified by column chromatography using chloroform-methanol (30:1) as the eluent to give 2-O-acetyllycorine-8,9-diphenol (**3a**) as a pale yellow powder (38.5 mg, yield 60%).

##### *α*-Dihydrolycorine (5)

A solution of lycorine hydrochloride (60 mg, 0.02 mmol) and 10% Pd/C (60 mg) in H_2_O (10 mL) was stirred under an atmosphere of H_2_ for 40 h to afford **4**. The solution was alkalized by NaOH (in solution, 1 M/L) and then filtered, and the cake was purified by column chromatography to afford α-dihydrolycorine (**5**) as a colourless solid (25 mg, yield 95%).

##### 5,7-dihydro-4H-[8,9]dioxolo[4,5-j]pyrrolo[3,2,1-de]phenanthridine (6)

A solution of lycorine (30 mg, 0.01 mmol) and Burgess reagent (24 mg, 0.1 mmol) in DMF (2 mL) was stirred under an atmosphere of H_2_ at 50 °C for 2 h to afford **4**. The solution was then evaporated to remove DMF and purified by column chromatography to afford 5,7-dihydro-4H-[8,9]dioxolo[4]pyrrolo[2,3]phenanthridine (**6**) as a colourless solid (20 mg, yield 80%).

##### 2-Oxolycorine (7)

Lycorine (30 mg, 0.01 mmol) was dissolved in 5 ml THF, to which Jones reagent (1 mL) was added. The mixture was then stirred at room temperature for 8 h, quenched with water (5 mL) and neutralized with NaHCO_3_. The reaction solution was evaporated to remove THF and extracted with CH_2_Cl_2_ (15 mL) twice. The organic layer was concentrated and purified by column chromatography to afford 2-oxolycorine (**7**) (18.5 mg, yield 65%).

##### Preparation of compounds 7a–c

Compound **7** (30 mg, 0.1 mmol) was dissolved in dry THF (10 mL), after which NaOH (10 mg, 0.25 mmol) and alkylating agents (methyl iodide or ethylbromide or allyl bromide, 1 mmol) were added. The mixture was stirred at room temperature for 24 h and then quenched with water (50 mL) in an ice bath. The reaction solution was evaporated to remove THF and extracted with CH_2_Cl_2_ (30 mL) twice. The organic layer was washed with saturated NaHCO_3_ and then brine, dried over MgSO_4_, filtered, and concentrated. The residue was purified by column chromatography to afford **7a**, **7b** and **7c**.

##### 1-methoxy-2-carbonyl-lycorine (7a)

6 mg, yield 20%.

##### 1-ethoxy-2-carbonyl-lycorine (7b)

9.4 mg, yield 30%.

1-allyloxy-2-carbonyl-lycorine (7c) 

6.6 mg, yield 20%.

### Bioactivity Assay

#### Cell Culture

Huh 7.5 human liver cells were cultured in Dulbecco’s modified Eagle medium (DMEM, Invitrogen, CA) supplemented with 10% inactivated foetal bovine serum (Invitrogen) and 1% penicillin-streptomycin (Invitrogen). Cells were digested with 0.05% trypsin-ethylene diamine tetraacetic acid (EDTA) (Invitrogen) and split twice a week.

#### HCV Infection and Treatment

The Huh 7.5 cells were seeded into 96-well plates (Costar) at a density of 3 × 10^4^ cells/cm^2^; after 24 hours of incubation, the cells were infected with HCV viral stock (chimeric HCV FL-J6/JFH/JC1 virus strain, approximately 45 IU/cell) and simultaneously treated with either one of the compounds in solution or with solvent only as a control. After 72 hours inoculation, the culture medium was removed and the intracellular RNA was extracted using the RNeasy Mini Kit (Qiagen). Additionally, Total intracellular proteins were also extracted using the Cyto-Buster Protein Extraction Reagent (Novagen) with a 1 mM protease inhibitor cocktail (Roche Applied Science). The intracellular levels of the HCV mRNA and the internal control gene glyceraldehyde-3-phosphate dehydrogenase (GAPDH) mRNA were quantified using an AgPath-ID™ One-Step RT-PCR Kit (Applied Biosystems). The fluorescent signal was detected using a 7500-fast real time PCR system (Applied Biosystems). The HCV core protein was detected by western blotting (see below).

#### Cytotoxicity Assay

50 *μ*L of Huh 7.5 cells at a density of 2 × 10^5^ cells/mL was plated into 96-well plates. Fresh culture medium containing test compounds at various concentrations was added 24 hours later. Cytotoxicity was evaluated using a tetrazolium (MTT) assay after 72 hours of treatment.

#### Western Blotting

The extracted total protein or viral lysates were denatured by adding loading buffer (5 × 250 mM tris-HCl, pH 6.8, 5% dithiothreitol, 10% SDS, 0.5% bromophenol blue, 50% glycerol), followed by boiling the lysates for 5 minutes at 100 °C. Proteins were analysed by SDS-PAGE and then transferred to nitrocellulose membranes using an electroblotter (Bio-Rad Laboratories). The membranes were blocked by 5% non-fat dry milk in TBS-T solution (20 mM Tris [pH 7.4], 150 mM NaCl, 0.1% Tween-20) for 1 hour and then washed three times for 10 minutes each in the TBS-T solution. Membrane samples were probed with a monoclonal antibody specific to the HCV core protein (diluted to 1 *μ*g/mL; Abcam, Ltd.) or Hsc70 (diluted to 0.2 *μ*g/mL; Santa Cruz Biotechnology, Inc., Santa Cruz, CA). As a control, probing experiments were performed using a polyclonal antibody against actin (diluted to 0.2 *μ*g/mL; Santa Cruz Biotechnology, Inc.). After being washed with TBS-T, the membranes were incubated for one hour at room temperature with the appropriate secondary antibody: goat anti-mouse for the HCV core protein, goat anti-rat for Hsc70, and goat anti-rabbit for actin (ZSGB-BIO, China). The protein signal was visualized and captured using Immobilon Western Chemiluminescent HRP Substrate ECL working solution (Millipore Inc.) and a ChemiDoc™ XRS gel imager system (Bio-Rad, CA).

## Additional Information

**How to cite this article**: Chen, D. *et al.* Design, Synthesis and Structure-Activity Relationship Optimization of Lycorine Derivatives for HCV Inhibition. *Sci. Rep.* **5**, 14972; doi: 10.1038/srep14972 (2015).

## Supplementary Material

Supplementary Information

## Figures and Tables

**Figure 1:**
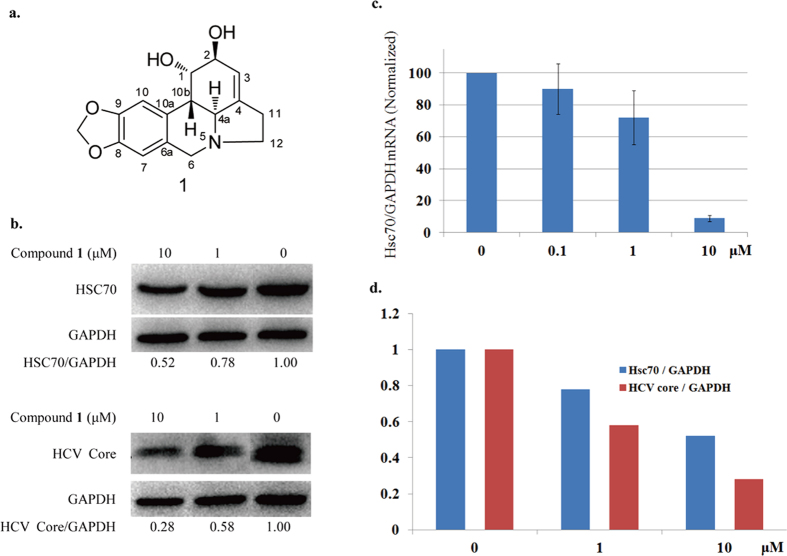
Hsc70 Down-regulation and anti-HCVactivity of lycorine. (**a**) The structure of lycorine; (**b**) The intracellular HCV core protein in Huh 7.5 cells decreased in a dose-dependent fashion following 72 h of treatment with compound 1 (lycorine) and the Hsc70 expression of Huh 7.5 cells also decreased; (**c**)The intracellular concentration of the Hsc70 mRNA; (**d**) The correlation between the inhibition of Hsc70 expression and the anti-HCV activity of lycorine. All mean and standard deviation data were obtained from quintuplicate experiments (N = 5).

**Table 1:** Anti-HCV activity of lycorine derivatives.

Comp.	Max Tested Concentration	^*a*^CC_50_ (*μ*M)	^*b*^EC_50_ (*μ*M)	^*c*^SI
lycorine	100 *μ*M	6.10 ± 0.17	0.316 ± 0.022	19
**1a**	100 *μ*M	23.55 ± 6.12	0.512 ± 0.493	46
**1b**	100 *μ*M	27.64 ± 1.38	1.342 ± 1.113	20.3
**1c**	100 *μ*M	10.98 ± 1.58	1.093 ± 0.141	10.4
**1d**	100 *μ*M	12.21 ± 3.41	1.018 ± 0.134	11.3
**2a**	100 *μ*M	>100 ± 0.00	>20	<5
**2b**	100 *μ*M	95.95 ± 17.72	>20	<4.5
**2c**	100 *μ*M	69.27 ± 1.58	13.9 ± 1.41	4.9
**3**	100 *μ*M	51.0 ± 8.1	0.24 ± 0.11	212
**3a**	100 *μ*M	49.2 ± 1.0	1.81 ± 0.45	27.2
**5**	100 *μ*M	>100	>20	<5
**6**	100 *μ*M	17.78 ± 1.41	3.236 ± 1.118	5.3
**7**	100 *μ*M	8.69 ± 0.60	0.11 ± 0.01	76.9
**7a**	100 *μ*M	>100 ± 0.00	2.23 ± 0.17	>44.8
**7b**	100 *μ*M	>100 ± 0.00	1.13 ± 0.07	>88.5
**7c**	100 *μ*M	>100 ± 0.00	1.28 ± 0.16	>78.3
VX-950	100 *μ*M	41.3 ± 4.9	0.16 ± 0.04	258

^*a*^CC_50_: minimum concentration required to kill at least 50% of the treated Huh 7.5 cells (relative to untreated controls). ^*b*^EC_50_: minimum concentration required to reduce HCV by at least 50% (relative to the rate in untreated control Huh 7.5 cells). ^*c*^SI = CC_50_/EC_50_.
