# Supplementary material for: Design, Synthesis and Structure-Activity Relationship Optimization of Lycorine Derivatives for HCV Inhibition
Source: Sci Rep. 2015 Oct 7;5:14972. doi: 10.1038/srep14972 (PMC4595722; doi:10.1038/srep14972)
Supplement: Supplementary Information [file srep14972-s1.pdf]

Design, Synthesis and Structure-Activity Relationship Optimization of Lycorine Derivatives for HCV Inhibition

Duozhi Chen, Jieyun Cai, Junjun Cheng, Chenxu Jing, Junlin Yin, Jiandong Jiang, Zonggen Peng and Xiaojiang Hao

S-1: Detailed reaction procedures of lycorine derivatives

S-1. Detailed reaction procedures of lycorine derivatives

1,2-Di-O-Acetyllycorine (**1a**). ^{1}H NMR (400 MHz, $CDCl_3$) δ: 6.73 (s, 1H), 6.59 (s, 1H), 5.92 (s, -OCH_2O-, 2H), 5.73 (s, 1H), 5.53 (m, 1H), 5.25 (m, 1H), 4.17 (d, J = 13.1 Hz, 1H), 3.56 (d, J =13.1 Hz, 1H), 3.33 (m, 1H), 2.87 (d, J = 10.2 Hz, 1H), 2.74 (d, J = 10.5 Hz, 1H), 2.62 (m, 2H), 2.44 (m, 1H), 2.07 (s, 3H), 1.94 (s, 3H); ^{13}C NMR (100 MHz, $CDCl_3$) δ: 170.1 (C), 169.9 (C), 146.4 (C), 146.2 (C), 146.1 (C), 129.5 (C), 126.5 (C), 113.8 (CH), 107.4 (CH), 105.0 (CH), 101.3 (-OCH_2O-), 70.9 (CH), 69.4 (CH), 61.2 (CH), 56.3 (CH_2), 53.2 (CH_2), 40.5 (CH), 28.9 (CH_2), 21.3 (CH_3), 20.9 (CH_3); HREIMS *m/z* 371.1360 $[M]^+$ (calcd for $C_{20}H_{21}NO_6$, 371.1369).

2-O-Acetyllycorine (1c). ^{1}H NMR (400 MHz, $CDCl_3$) δ: 6.80 (s, 1H), 6.62 (s, 1H), 5.94-5.93 (m, 2H), 5.48 (m, 1H), 5.35 (m, 1H), 4.49 (m, 1H), 4.13 (d, J = 14.3 Hz, 1H), 3.54 (d, J = 14.3 Hz, 1H), 3.37 (s, 1H), 2.79 (d, J = 9.1 Hz, 1H), 2.71 (d, J = 9.1 Hz, 1H), 2.65 (m, 2H), 2.38 (m, 1H), 2.08 (s, 3H); ^{13}C NMR (100 MHz, $CDCl_3$) δ: 170.6 (C), 146.6 (C), 146.3 (C), 145.8 (C), 129.8 (C), 127.2 (C), 113.9 (CH), 107.5 (CH), 104.7 (CH), 101.1 (CH_2), 73.6 (CH), 69.0 (CH), 60.6 (CH), 58.4(CH_2), 57.2(CH_2), 41.5 (CH), 28.8 (CH_2), 21.2 (CH_3); HREIMS *m/z* 329.1254 $[M]^+$ (calcd for $C_{18}H_{19}NO_5$, 329.1263).

1-O-Acetyllycorine (1d). ^{1}H NMR (500MHz, $CDCl_3$) δ: 6.69 (s, 1H), 6.45 (s, 1H), 5.92 (s, 2H), 5.58 (m, 1H), 5.50 (m, 1H), 4.18 (m, 1H), 4.15 (d, J = 14.0 Hz, 1H), 3.49 (d, J = 14.0 Hz, 1H), 3.33 (m, 1H), 2.86 -2.85 (m, 2H), 2.61 (m, 2H), 2.36 (m, 1H), 1.95 (s, 3H); ^{13}C NMR (125 MHz, $CDCl_3$) δ: 171.8 (C), 146.5 (C), 146.2 (C), 143.5 (C), 129.3 (C), 127.4 (C)，117.4(CH), 107.2 (CH), 104.9 (CH), 101.3 (CH_2), 72.9 (CH), 68.9 (CH), 61.8 (CH), 56.8 (CH_2), 53.7 (CH_2), 39.4 (CH), 28.4 (CH_2), 21.0(CH_3). HREIMS *m/z* 329.1251 $[M]^+$ (calcd for $C_{18}H_{19}NO_5$, 329.1263).

1,2-Di-O-Acetyl-6-carbonyl-lycorine (2b). ^{1}H NMR (400 MHz, $CDCl_3$) δ: 7.55 (s, 1H), 6.68 (s, 1H), 6.00 (s, 2H), 5.73 (m, 1H), 5.61 (m, 1H), 5.27 (s, 1H), 4.23 (d, J = 12.5 Hz, 1H), 3.91-3.71 (m, 2H), 3.03 (d, J = 12.5 Hz, 1H), 2.86-2.75 (m, 2H), 2.07 (s, 3H), 2.01 (s, 3H); ^{13}C NMR (100 MHz, $CDCl_3$) δ: 169.8 (C), 169.4 (C), 162.5 (C), 150.7 (C), 146.9 (C), 143.6 (C), 131.8 (C), 126.2 (C), 115.4 (CH), 108.9 (CH), 103.5 (CH), 101.7 (CH_2), 70.1 (CH), 67.3 (CH), 55.1 (CH), 43.5 (CH_2), 40.3 (CH), 28.5 (CH_2), 20.9 (CH_3), 20.8 (CH_3). HREIMS *m/z* 385.1170 $[M]^+$ (calcd for $C_{20}H_{19}NO_7$, 385.1162).

6-Carbonyllycorine (2a). ^{1}H NMR (400 MHz, $CDCl_3$) δ: 7.34 (s, 1H), 6.63 (s, 1H), 5.78 (s, 2H), 5.43(s, 1H), 4.32 (m, 1H), 4.26 (m, 1H), 3.64-3.47 (m, 2H), 3.14-2.88 (m, 2H), 2.58-2.49 (m, 2H)； ^{13}C NMR (100 MHz, $CDCl_3$) δ: 162.7 (C), 150.4 (C), 146.6 (C), 142.0 (C), 133.5 (C), 126.5 (C), 116.8 (CH), 108.7 (CH), 103.5 (CH), 101.5

(CH_2), 75.6 (CH), 75.5 (CH), 57.6 (CH_3), 57.5 (CH_3), 55.1 (CH), 43.4 (CH_2), 41.3 (CH), 28.5 (CH_2); HREIMS *m/z* 301.0962 $[M]^+$ (calcd for $C_{16}H_{15}NO_5$, 301.0950).

1,2-Di-O-Methyl-6-carbonyllycorine (2c). ^1H NMR (400 MHz, $CDCl_3$) δ: 7.57 (s, 1H), 6.73 (s, 1H), 6.06 (dd, *J* = 4.3 Hz, 1.1 Hz, 2H), 5.69 (s, 1H), 4.13 (d, *J* = 7.5 Hz, 1H), 4.07 (s, 1H), 3.96 (1H, s), 3.80-3.75 (m, 2H), 3.64 (s, 3H), 3.57 (s, 3H), 2.88 (, *J* = 12.3 Hz, 1H), 2.76 (m, 2H)； ^{13}C NMR (100 MHz, $CDCl_3$) δ: 162.7 (C), 150.9 (C), 146.8 (C), 142.0 (C), 133.1 (C), 126.1 (C), 116.8 (CH), 108.1 (CH), 103.0 (CH), 101.5 (CH_2), 75.3 (CH), 75.5 (CH), 57.6 (CH_3), 57.5 (CH_3), 55.1 (CH), 43.4 (CH_2), 41.3 (CH), 28.5 (CH_2); HREIMS *m/z* 329.1273 $[M]^+$ (calcd for $C_{18}H_{19}NO_5$, 329.1263).

1,2-Di-O-Methyllycorine (1b). ^1H NMR (400 MHz, $CDCl_3$) δ: 6.83 (s, 1H), 6.61 (s, 1H), 5.91 (dd, *J* = 6.4, 1.3 Hz, 2H), 5.70 (m, 1H), 4.10-4.07 (m, 2H), 3.99-3.91 (m, 2H), 3.56 (s, 3H), 3.43 (s, 3H), 3.29-3.17(m, 2H), 2.72 (dd, *J* = 21.9, 10.6 Hz, 2H), 2.52 (dd, *J* = 21.3, 18.5 Hz, 2H)； ^{13}C NMR (100 MHz, $CDCl_3$) δ: 146.7 (C), 145.3 (C), 144.6 (C), 130.4 (C), 128.4 (C), 115.2 (CH), 107.6 (CH), 104.9 (CH), 100.3 (CH_2), 71.5 (CH), 61.1 (CH), 58.0 (CH_3), 57.2 (CH_3), 56.8(CH_2), 53.6 (CH), 51.5 (CH_2), 41.6 (CH), 28.7 (CH_2); HREIMS *m/z* 315.1473 $[M]^+$ (calcd for $C_{18}H_{21}NO_4$, 315.1471).

1,2-Di-O-Acetyllycorine-8,9-diphenol (3). ^{1}H NMR (500 MHz, CD_3OD) δ 6.73 (s, 1H), 6.42 (s, 1H), 5.95 - 5.82 (m, 1H), 5.78 - 5.66 (m, 1H), 5.64 - 5.45 (m, 1H), 4.16 - 3.99 (m, 1H), 3.68 - 3.50 (m, 1H), 3.48 - 3.31 (m, 1H), 3.29 - 2.96 (m, 2H), 2.65 - 2.25 (m, 3H), 2.09 (s, 3H), 2.02 (s, 3H); ^{13}C NMR (125 MHz, CD_3OD) δ 173.2, 172.6, 149.7, 143.8, 142.4, 125.7, 125.2, 114.7, 112.8, 113.4, 73.1, 69.7, 61.9, 58.5, 51.7, 37.5, 30.9, 22.1; HREIMS *m/z* 359.1380 $[M]^+$ (calcd for $C_{19}H_{21}NO_6$, 359.1369).

2-O-Acetyllycorine-8,9-diphenol (3a). ^{1}H NMR (500 MHz, DMSO-d_6) δ 6.72 (s, 1H), 6.65 (s, 1H), 5.67 (s, 1H), 5.57 (s, 1H), 4.54 (s, 1H), 4.07 (s, 1H), 3.62 (s, 1H), 3.28 - 2.95 (m, 3H), 2.50 (s, 1H), 2.33 (s, 2H), 2.09 (s, 3H); ^{13}C NMR (125 MHz, DMSO-d_6) δ 170.3, 149.4, 144.4, 141.8, 126.9, 125.3, 115.5, 113.3, 112.9, 71.9, 70.6, 63.4, 57.9, 51.0, 41.9, 30.1, 21.2; HREIMS *m/z* 317.1273 $[M]^+$ (calcd for $C_{17}H_{19}NO_5$, 317.1263).

***α*-Dihydrolycorine (5).** ^{1}H NMR (400 MHz, DMSO-d_6) δ: 7.06 (1H, s), 6.83 (1H,s), 5.93 (2H, d, J = 4.3), 4.58 (1H, d, J = 13.7), 4.49 (1H, s), 4.08 (1H, d, J = 13.9 Hz), 4.01 (1H, dd, J = 6.5, 3.5 Hz), 3.59 (1H, overlap), 3.58 (1H, overlap), 3.42 (1H, dd, J = 11.1, 8.0 Hz), 3.24 (1H, d, J = 12.0 Hz), 2.71-2.58 (1H, m), 2.52 (1H, dt, J = 12.5, 6.0 Hz), 2.24 (1H, ddd, J = 15.1, 6.7, 3.9 Hz),2.14 (1H, dt, J = 12.5, 6.0 Hz), 1.93 (1H, d, J = 15.1 Hz); ^{13}C NMR (100 MHz, $CDCl_3$) δ: 149.9 (C), 147.8 (C), 133.3 (C), 124.3 (C), 108.5 (CH), 107.3 (CH), 102.7 (CH_2), 70.2 (CH), 69.5 (CH), 65.0 (CH),

56.7 (CH_2), 54.1 (CH_2), 37.0 (CH), 33.1 (CH), 30.7 (CH_2), 27.7 (CH_2); HREIMS *m/z* 289.1319$[M]^+$ (calcd for $C_{16}H_{19}NO_4$, 289.1314).

5,7-dihydro-4H-[8,9]dioxolo[4,5-j]pyrrolo[3,2,1-de]phenanthridine (6). ^{1}H NMR (400 MHz, $CDCl_3$) δ: 7.23 (1H, t, J = 5.3 Hz,), 7.22 (1H, d, J = 9.4 Hz), 7.01 (1H, d, J = 7.3 Hz), 6.79 (1H, t, J = 7.4 Hz), 6.61 (1H, s), 5.98 (2H, s), 4.03 (2H, s), 3.32 (2H, t, J = 7.9 Hz), 3.01 (2H, t, J = 8.3 Hz), 2.08 (1H, s), 1.36–1.20 (1H, m); ^{13}C NMR (100 MHz, $CDCl_3$) δ: 150.3(C), 147.8 (C), 146.2 (C), 129.0 (C), 128.4 (C), 126.6 (C), 123.2 (C), 123.7 (CH), 119.8 (CH), 119.5 (CH), 119.2 (CH), 107.6 (CH), 102.2 (CH), 101.0 (CH_2), 55.4 (CH_2), 53.7 (CH_2), 29.0 (CH_2); HREIMS *m/z* 251.0950$[M]^+$ (calcd for $C_{16}H_{13}NO_2$, 251.0946).

2-carbonyl-lycorine (7). ^{1}H NMR (400 MHz, $CDCl_3$) δ 6.75 (s, 1H), 6.59 (s, 1H), 5.88 (s, 2H), 5.86 (s, 1H), 4.46 (d, J = 2.9 Hz, 1H), 4.09 (d, J = 14.1 Hz, 1H), 3.54 (d, J = 14.0 Hz, 1H), 3.45-3.32 (m, 1H), 3.21 (dd, J = 44.5, 9.8 Hz, 2H), 2.89-2.82 (m, 2H), 2.44 (dd, J = 17.2, 8.6 Hz, 1H); ^{13}C NMR (100 MHz, $CDCl_3$) δ 195.4, 169.5, 145.5, 145.7, 130.5, 125.9, 119.6, 107.5, 104.9, 103.3, 70.5, 62.7, 54.2, 53.6, 46.7, 31.1; HREIMS *m/z* 285.1003 $[M]^+$ (calcd for $C_{16}H_{15}NO_4$, 285.1001).

1 -methoxy-2-carbonyl-lycorine (7a). ^{1}H NMR (500 MHz, $CDCl_3$) δ 6.68 (s, 1H), 6.52 (s, 1H), 6.03-5.96 (overlap, 3H), 4.49-4.39 (m, 1H), 4.30-4.20 (m, 1H), 4.00-3.89

(m, 1H), 3.76-3.61 (m, 2H), 3.61 (s, 3H), 3.36-3.28 (m, 1H), 2.63-2.45 (m, 3H); ^{13}C NMR (125 MHz, $CDCl_3$) δ 194.8, 164.5, 145.6, 145.2, 129.3, 127.2, 116.2, 107.4, 106.1, 101.6, 84.7, 60.3, 58.5, 56.8, 51.0, 41.5, 30.9; HREIMS *m/z* 299.1166 $[M]^+$ (calcd for $C_{17}H_{17}NO_4$, 299.1158).

1- ethoxy -2-carbonyl-lycorine (7b). ^{1}H NMR (500 MHz, $CDCl_3$) δ 6.72 (s, 1H), 6.64 (s, 1H), 6.06-5.92 (overlap, 3H), 4.43 (m, 1H), 4.34-4.18 (m, 1H), 4.02-3.94 (m, 1H), 3.91-3.83 (m, 2H), 3.76-3.63 (m, 2H), 3.41-3.26 (m, 1H), 2.64-2.47 (m, 3H), 1.27-1.06 (m, 3H); ^{13}C NMR (125 MHz, $CDCl_3$) δ 196.2, 163.7, 146.7, 144.7, 129.4, 126.9, 115.3, 107.8, 106.4, 102.1, 82.6, 65.1, 61.2, 56.2, 51.3, 40.7, 31.2, 16.1. HREIMS *m/z* 313.1310 $[M]^+$ (calcd for $C_{18}H_{19}NO_4$, 313.1314).

1- allyloxy-2-carbonyl-lycorine (7c). ^{1}H NMR (500 MHz, $CDCl_3$) δ 6.78 (s, 1H), 6.64 (s, 1H), 6.18-5.93 (m, 4H), 5.51-5.35 (m, 1H), 5.34-5.25 (m, 1H), 4.76-4.64 (m, 1H), 4.26 (d, 1H), 4.21-4.12 (m, 2H), 4.06-3.93 (m, 1H), 3.79-3.73 (m, 1H), 3.72-3.66 (m, 1H), 3.45-3.23 (m, 1H), 2.67-2.45 (m, 3H); ^{13}C NMR (125 MHz, $CDCl_3$) δ 195.8, 163.6, 145.7, 144.8, 134.6, 129.5, 126.7, 116.8, 115.8, 107.6, 105.8, 102.9, 83.1, 73.8, 61.0, 58.3, 52.5, 41.6, 30.1; HREIMS *m/z* 325.1316 $[M]^+$ (calcd for $C_{19}H_{19}NO_4$, 325.1314).
